# VRrec: A zero-coding open-source VR body-motion capture tool for behavioral research

**DOI:** 10.3758/s13428-026-03169-9

**Published:** 2026-09-30

**Authors:** Pawel Kobylinski, Bartosz Muczynski, Daniel Cnotkowski, Mariusz Wierzbowski, Cezary Biele

**Affiliations:** 1 National Information Processing Institute, al. Niepodleglosci 188 B, 00-608 Warsaw, Poland; 2 Maritime University of Szczecin, Szczecin, Poland; 3 VIZJA University, Warsaw, Poland

**Keywords:** Behavioral research tool, Virtual reality, Body-motion capture, Open source

## Abstract

We present VRrec, an open-source, motion-tracking and recording tool for PC-based virtual reality (VR). The tool addresses a persistent gap in VR-based experimental research by enabling real-time capture of head, hand, and optional body-tracking data without the need to code. VRrec runs discreetly in the background, collecting positional and rotational data as researchers engage participants with third-party VR applications. This design lets researchers use flexible paradigms that leverage off-the-shelf VR content, eliminating the need to build or modify custom software. However, because VRrec does not read application-level events or object states from the target VR software, it does not directly support paradigms that require synchronization with in-app events or distances to dynamic virtual objects or avatars. VRrec still allows manual event marking for contextual annotation. The resulting data are stored as plain text, which can be imported into various analysis pipelines to derive behavioral metrics in fields such as social psychology, rehabilitation, and consumer research. Evaluations show consistent and stable recording performance with minimal impact on system resources, while also indicating some limitations for study designs that require millisecond-accurate timing precision. By streamlining VR motion data acquisition, VRrec broadens the range of feasible VR study designs and reduces technical barriers, making VR-based behavioral research more accessible. Both the source code and the executable version of VRrec are freely available on GitHub.

## Introduction

### Motivation

Virtual reality (VR) technology has opened new avenues for exploring human behavior, providing researchers with the opportunity to study a variety of phenomena in controlled, immersive environments (Cipresso, Giglioli, Raya, & Riva, 2018; Pan & Hamilton, 2018; Rizzo, Goodwin, De Vito, & Bell, 2021). Modern six-degree-of-freedom (6DOF) VR systems enable body-motion tracking (Caserman, Garcia-Agundez, Konrad, Göbel, & Steinmetz, 2019; Gonçalves, Melo, Barbosa, Vasconcelos-Raposo, & Bessa, 2022), which allows not only for enhanced immersion, embodiment, and interaction (Alcañiz Raya et al., 2020; Gonzalez-Franco & Lanier, 2017; Jerald, 2015; Ruddle & Lessels, 2009) but also for body-motion data recording and – consequently – body-motion data analysis, opening the possibility for researchers to gain insights into various aspects of human behavior, cognition, interactions within customizable environments, and human body motion itself (Kloiber, Collins, Eggeling, Preiner, & Schreck, 2022; Miller, Herrera, Jun, Landay, & Bailenson, 2020; Pan & Hamilton, 2018; Yaremych & Persky, 2019). In line with the advances in VR technology and its applications for exploring human behavior – as well as the opportunities it offers for body-motion tracking and data analysis – we present VRrec, an open-source body-motion tracking and recording tool for PC-based VR, designed to operate discreetly in the background to collect motion data while researchers engage participants with any VR application of their choosing, thereby providing a versatile, user-friendly solution for capturing body-motion information across diverse virtual environments and scenarios.

In behavioral research that uses VR, solutions offering body-motion recording functionality are typically designed as an all-in-one system, in which the same program delivers the VR experience and captures motion data (Borgnis et al., 2022; Pieri, Tosi, & Romano, 2023; Yaremych & Persky, 2019). This design strategy requires a customized application for each experiment, one that displays the virtual environment and facilitates body-motion data acquisition. VRrec circumvents this requirement, allowing researchers to design studies with off-the-shelf PC-based VR applications and thereby offering a zero-coding solution in the strict sense that researchers need neither to write code nor to build or rebuild the target VR application in order to capture body-motion data. Driven by a desire to empower researchers, our motivation is to provide this background-capture solution, simplifying the design and implementation of VR-based experiments, particularly when resources are limited, or a comprehensive all-in-one system is unnecessary, or when the research question concerns behavior in mature third-party VR applications.

It is crucial to recognize that the proposed approach, in contrast to VR applications explicitly crafted for specific experimental purposes, entails a trade-off. VRrec cannot directly interact with the application managing the virtual environment and, as such, remains uninformed about the ongoing application-level events and object states within the experiment. Nevertheless, VRrec partially compensates for this limitation by enabling event marking by an observer or experimenter through a computer keyboard.

VRrec records body-motion data in a text file but, in accordance with our assumptions, it is not intended as a data-analysis tool and does not encompass data-analysis functionality. In paragraph “VRrec data output”, we provide an overview of how to decipher the data produced by our application, shedding light on the key aspects of understanding body-motion data and thus providing researchers with a foundation on which to build for their specific analysis needs.

It is essential to highlight that the data recorded by VRrec, in and of itself, does not represent specific indicators of any psychological constructs. This is because body-motion data primarily consist of raw, context-free information about the participant’s movements within the virtual environment. These data points alone lack the context necessary to provide meaningful insights into psychological phenomena. However, VRrec’s recorded data lets researchers construct indicators closely aligned with the theoretical framework of a particular experiment or observational study. By combining VRrec’s body-motion data with the context provided by the research design and experimental conditions, researchers can extract meaningful information and develop metrics relevant to their specific research questions. This approach enables a nuanced understanding of human behavior, cognition, and interactions within the virtual environment, tailored to each study’s objectives. In the following paragraphs (“Body-motion capture in behavioral research” and “Body-motion capture in behavioral research”), we provide a brief overview of examples illustrating this strategy of combining body-motion data with the context of research design and experimental conditions. First, we present examples from research conducted in non-VR settings, followed by those utilizing VR technology. This review aims to demonstrate the versatility and applicability of the proposed strategy in various research settings.

### Body-motion capture in behavioral research

Growing interest in human behavior and its underlying mechanisms has increased demand for more precise and accurate methods to study body movements. Motion-capture technology, with its ability to provide a detailed and objective representation of body motion, has emerged as a valuable tool for behavioral research. In this section, we discuss some of the most notable applications of motion capture technology in behavioral research, highlighting the versatility and potential of this approach.

One of the most well-established applications of motion capture in behavioral research is studying social interactions and interpersonal dynamics (Holler & Beattie, 2005). By analyzing body movement, researchers can gain insight into non-verbal communication, such as gestures, posture, and facial expressions, which play a crucial role in conveying emotions and intentions (Pollick, Lestou, Ryu, & Cho, 2002). By examining the subtle nuances of body language, motion capture can provide a deeper understanding of the factors influencing human interaction, enabling researchers to investigate topics such as empathy (Carlson, Burger, & Toiviainen, 2019) and romantic relationships (Preissmann et al., 2016).

Another significant application of motion-capture technology in behavioral research is the investigation of motor-skill development and rehabilitation (Cirstea, Ptito, & Levin, 2006). By monitoring and quantifying body movements, researchers can study the acquisition of new skills, the impact of different training methodologies, and the effectiveness of motion capture in educational research (for a review, see: Reuter and Schindler (2023)). Motion capture has been particularly useful in the fields of sports science (van der Kruk & Reijne, 2018), occupational therapy (Winiarski, Molek-Winiarska, Chomatowska, Sipko, & Dyvak, 2021), and physiotherapy (Cannell et al., 2018), where understanding the intricacies of movement patterns is essential for optimizing performance and preventing injury.

Emotion recognition and expression are another important area where motion capture has proven to be a valuable tool (Dittrich, Troscianko, Lea, & Morgan, 1996). By extracting features from body movements, researchers can develop models to classify emotions and assess emotional states (Castellano, Villalba, & Camurri, 2007). This knowledge can then be applied to fields like psychology, where understanding affective responses is crucial for the diagnosis and treatment of mental health disorders (Gross, Uusberg, & Uusberg, 2019), or human–computer interaction, where emotion recognition can enhance the development of more empathetic and responsive technologies (Kapoor & Verma, 2024).

Furthermore, researchers have used motion capture alongside other techniques to explore the relationship between body motion and other behavioral aspects. By integrating motion-capture data with physiological measures (e.g., electrophysiological measures), researchers can obtain a more comprehensive picture of human behavior (Pradhan, Engineer, Nadin, & Prabhakaran, 2007). This multimodal approach allows for the examination of complex interactions between different aspects of behavior, leading to a richer and more nuanced understanding of the factors that influence human actions.

In summary, the applications of motion capture technology in behavioral research are vast and varied. From social interactions to motor skill development, emotion recognition, and beyond, motion capture has the potential to transform our understanding of human behavior by providing accurate, objective, and detailed insights into body movement. As technology continues to advance, the scope and impact of motion capture in behavioral research are expected to grow, especially in rapidly developing areas such as virtual and augmented reality (Miyakoshi, Gehrke, Gramann, Makeig, & Iversen, 2021).

### Body-motion capture in VR behavioral research

Slater, Steed, McCarthy, and Maringelli (1998) conducted an experiment to investigate the impact of body movements on presence within a virtual environment. Participants walked through a virtual field of trees and counted those with diseased leaves, with tree-height variation and task complexity as the two factors assessed. The findings demonstrated a significant positive correlation between reported presence and the amount of body movement, particularly in head yaw (horizontal rotation of the head) and the degree to which participants bent down and stood up. This classic VR study in experimental psychology highlights the importance of whole-body movement in enhancing presence in virtual environments.

In another classic study, Bailenson, Blascovich, Beall, and Loomis (2001) used immersive virtual-environment technology to examine proxemics (Hall et al., 1968) – i.e., spacing behavior in social interactions. Participants were immersed in a virtual room with an embodied agent and instructed to walk toward and around the agent under the pretext of a memory task. The virtual-environment system automatically tracked the distance between the participant and the agent. The results revealed that participants maintained more space around agents than around similarly sized non-human objects; female participants kept a greater interpersonal distance when the agent engaged them in eye contact, whereas male participants did not. These findings demonstrate the potential of using body-motion data and immersive virtual-environment technology in the study of proxemics. In line with the proxemics framework, Hasler and Friedman (2012) investigated the potential of using virtual worlds for cross-cultural and intercultural communication research. They examined differences in spatial social behavior between Asian and European cultures during dyadic avatar interactions. The results showed that Asian dyads interacted at larger distances than European dyads, consistent with the cross-cultural differences typically observed in face-to-face interactions. By tracking and analyzing avatar locomotion, the study demonstrates the utility of virtual reality and body-motion data in conducting cross-cultural research.

Applying the proxemics paradigm to the examination of implicit associations and prejudice, Dotsch and Wigboldus (2008) investigated how prejudiced implicit associations affect physiological and automatic behavioral responses in social interactions. Native Dutch participants were immersed in a virtual environment and encountered avatars with either White or Moroccan facial features. Researchers tracked participants’ positions and found that they maintained greater distance and showed higher skin conductance when approaching Moroccan avatars than White avatars. Implicit negative associations with Moroccans moderated both effects, and skin-conductance-level effects fully mediated the relationship between implicit prejudice and distance effects. This study demonstrates the significance of using body-motion data in VR-based experimental psychology research to better understand how prejudiced implicit associations may unintentionally lead to impulsive discriminatory responses. Further exploring the potential of body-motion data analysis to study prejudice, Peck, Good, and Seitz (2021) investigated shooter and racial bias in an immersive virtual environment. Although latency to shoot did not differ significantly by race or socioeconomic status, more nuanced head-motion analysis predicted participants’ implicit racism scores, and hand-motion analysis predicted shooting-accuracy bias. By utilizing body-motion data analysis, the study offers a fresh perspective on racial-bias research that goes beyond the proxemics framework.

Both proxemics and social-prejudice concepts resonate with a theoretical exploration by Jeong, Kim, Xu, and Miller (2021), in which the authors analyze the use of body-motion data in VR with respect to the Proteus effect (Yee, Bailenson, & Ducheneaut, 2009). The effect implies that identity cues embedded in user avatars can trigger changes in both attitudes and behaviors. The authors note that it has been examined in diverse contexts, including gender-based avatar personalization, educational stereotype threat, gender-specific stereotypical actions, sexual self-objectification, dating, pedagogy, consumer preferences, public-speaking anxiety, and portrayals of older bodies. Notably, the authors connect this concept to approach-avoidance conflict, a foundational theoretical framework in social psychology, first proposed by Miller and Dollard (Miller, 1944; Miller & Dollard, 1945).

Another theoretical exploration, proposed by Bacos and Carroll (2018), centers on how body-motion data, captured via VR, can shed light on social cognition in e-learning settings. Drawing on the self-other equivalence framework, they argue that greater movement similarity between individuals fosters shared motor and visual experiences, thereby enhancing the processes of perceiving, predicting, and interpreting others’ behavior. This line of reasoning highlights the potential of body-motion data analysis in understanding and enhancing e-learning experiences. Interestingly, Kobylinski, Pochwatko, & Biele (2019) show that employing distance-based metrics can help quantify inter-subject similarities in visual attention and spatial behavior in VR contexts, thereby reinforcing the possibility of analyzing body-motion similarity to understand shared experiences.

Building on insights into how body-motion data can deepen our understanding of social interactions, Gürerk, Bönsch, Kittsteiner, and Staffeldt (2018) used VR technology to create a naturalistic work setting in an immersive virtual environment. In this study, human participants performed a sorting task alongside a computer-generated virtual human coworker whose productivity level was experimentally manipulated. Although the coworker’s productivity had no overall effect on participants’ work output, individuals with a more competitive disposition performed significantly better when their virtual partner was highly productive. By analyzing hand- and head-motion data, the authors demonstrated that these competitive participants approached the task with greater care, illustrating the value of body-motion analysis for exploring workplace dynamics in VR.

In a different domain of behavioral research, Cavedoni, Chirico, Pedroli, Cipresso, and Riva (2020) introduced a perspective recommending an integrated approach that combines VR and artificial intelligence to facilitate earlier detection of mild cognitive impairment (MCI) in older adults. They describe how controlled virtual simulations of daily activities can enable clinicians to record patient kinematics (particularly gait) during tasks that simulate instrumental activities of daily living. Machine learning algorithms can then analyze these body-motion data alongside clinical and neuropsychological measures, potentially yielding a predictive model that identifies subtle motor and cognitive impairments in MCI. Their perspective not only underscores the promise of VR in clinical settings but also illustrates the broader potential of body-motion data to inform diagnostic and therapeutic interventions.

The most recent studies have confirmed the usefulness of VR motion data in diverse behavioral research contexts. For instance, in a clinical application, Oh et al. (2024) developed and tested a VR-based diagnostic tool that captured movement patterns (headset and controller tracking, reaction times, and distractibility metrics) in children with and without ADHD. They implemented everyday activities in VR (e.g., organizing a room, packing a backpack, solving puzzles), then fed these behavioral data into a machine-learning model. Their system achieved high diagnostic accuracy in distinguishing ADHD from non-ADHD participants, highlighting how kinematic and performance data can complement subjective assessments by parents or clinicians. Importantly, capturing head and hand motion provided objective indicators of inattention, hyperactivity, and impulsivity, showing that VR body-motion data can yield powerful insights not only in classical social or motor-skill research but also in clinical and diagnostic applications.

Another recent illustration of how VR body-motion data can reveal psychological states comes from Crawford et al. (2024), who focused on emotions, showing that fear responses are discernible from VR body-motion cues even in minimal avatar displays. They recorded the body movements of participants walking a plank in VR set either at ground level or at a dizzying 80-story height to induce fear. Motion-capture data were saved and later presented as simple point-light-style animations. Observers who viewed these motion-only replays accurately distinguished high-fear walks from non-fearful ones and even rated the intensity of the individuals’ fear similarly to the individuals’ own self-reports. This finding underscores the potency of VR motion data for research on emotion expression and perception.

The examples highlighted in this section illustrate the diverse range of research questions and applications that VR body-motion data can address, from social interaction and prejudice research to clinical diagnostics and beyond. Our aim here has not been to provide a comprehensive review of all relevant findings or theoretical frameworks; rather, we have offered an illustrative glimpse into the breadth of possibilities. By showing how body movement can serve as a valuable behavioral indicator across numerous contexts, we hope to inspire new investigations that leverage VR motion capture – particularly through tools such as VRrec – to study psychological constructs and research paradigms of many kinds.

## State of the art

Recording body movements is of considerable importance to the scientific community; however, it is an even more desirable feature in the computer-graphics industry, particularly for creating complex human animations used in video games and animated films. A variety of specialized hardware solutions exist for precise body tracking, many of which can be integrated into VR systems. Examples include OptiTrack (NaturalPoint, Inc., 2025) and Polhemus (Polhemus, Inc., 2025); however, these hardware-based approaches fall outside the scope of this paper and will not be discussed further. Instead, the following section focuses on software-based solutions that utilize consumer-grade VR hardware.

Two general approaches exist for recording movement data in VR applications. The first involves embedding the tracking functionality directly into the application itself. The second relies on external software that captures and logs movement data independently. The former is only feasible when developing proprietary applications or when full access to and permission to modify the source code are granted – an often limiting condition that precludes analysis of user movement within popular commercial applications. This method also requires programming expertise and familiarity with development environments such as Unity (Unity Technologies, 2025), Unreal Engine (Epic Games, Inc., 2025), or Vizard (WorldViz, Inc., 2025). Furthermore, software embedded in specific engine versions may become incompatible with future updates.

Despite these limitations, open-source tools and plug-ins for such platforms have already been developed and are available for integration into ongoing projects. These tools employ varying approaches. A basic example involves detecting whether a user is located within a predefined hotspot, recording the duration spent in that area, and visualizing the data via heatmaps (Šimkovič, Zajíček, & Hajtmanek, 2019). This concept was expanded into a Unity plug-in, enabling hotspot creation and data recording within the Unity Editor without requiring programming knowledge (de Paiva Guimarães et al., 2021). REC (Gorisse et al., 2022) offers a more advanced solution, capturing positional data from an animated 3D avatar that mirrors real-time movement through VR hardware or other body-tracking systems, but it cannot run motion-capture recording in the background while a separate application is active. ManySense VR (Moon, Jeong, Oh, Laine, & Seo, 2022) represents a more comprehensive platform, capable of recording not only movement data but also input from other sensors and peripherals, including eye trackers, facial trackers, EEGs, and heart rate monitors. However, it too must be embedded within a custom Unity application. Additional examples include the Unreal-Engine-based implementation by Kloiber et al. (2020) and a dedicated tool developed for the Vizard platform by Schuetz, Karimpur, and Fiehler (2023).

Brekel OpenVR Recorder (Brekel 3D, 2017) is a fully standalone commercial application for recording VR movement data independently of the application being explored. It was developed for Microsoft Windows and supports the OpenXR API (Khronos Group, 2025), the HTC Vive (HTC Corporation, 2018) and Oculus (Meta Platforms Technologies, LLC, 2025) device families. It allows users to save the data as either an “.fbx” or a text file. While very capable, it lacks options to customize how it works, which data it saves, or the format. Unlike VRrec – which streams each sample straight to disk in real time, so an unexpected crash never jeopardizes the data already collected – Brekel OpenVR Recorder buffers motion-capture data in RAM, which means that if the program crashes, all unsaved data are lost. Mocap Fusion [VR] (Animation Prep Studios, 2023) is another commercial program that promises similar functionality, but at the time of our paper’s submission it offered only animation-data export.

PLUME (Javerliat, Villenave, Raimbaud, & Lavoué, 2024) and the Virtual Reality Scientific Toolkit (VRSTK) (Deuchler, Hettmann, Hepperle, & Wölfel, 2023) are both comprehensive open-source toolboxes designed for recording and analyzing user behavior and physiological signals in XR applications developed with Unity. While both tools are open-source, support standardized data formats, and enable synchronized acquisition of movement data, they share a critical limitation: they are tightly coupled to the Unity engine and must be integrated during application development, preventing capture of motion data from third-party VR applications.

The Unity Experiment Framework (UXF) (Brookes, Warburton, Alghadier, Mon-Williams, & Mushtaq, 2020) is another important related solution. UXF is a software framework for developing behavioral experiments within Unity, providing an experiment structure based on sessions, blocks, and trials and supporting continuous data collection such as position and rotation tracking. However, UXF is intended to be integrated into the Unity project that implements the experiment, rather than to record motion unobtrusively from already-built third-party PC-based VR applications.

A similar distinction applies to more specialized Unity-based toolkits such as VRThreat (Brookes, Hall, Frühholz, & Bach, 2024) and the CogLearn Toolkit for Unity (Rodriguez Lopez, Liu, Mancinelli, Brookes, & Bach, 2025). CogLearn explicitly uses UXF for trial management and data collection, and both solutions are designed for Unity-centered experimental workflows rather than for external recording of arbitrary mature third-party VR applications. They may reduce the technical burden within specific paradigms, but unlike VRrec, they do not target background motion capture from already-built VR software without direct application integration or rebuilding the target application.

## VRrec details

### System requirements and compatibility

In its current implementation, VRrec is a Windows desktop application written in C# for .NET 6 or higher. From the user’s perspective, running VRrec requires a Windows PC, the .NET 6 runtime (or higher), and a working SteamVR installation configured for the target PC-VR setup. Technically, VRrec uses the OpenVR SDK (Valve Corporation, 2026a) and relies on the SteamVR runtime (Valve Corporation, 2023) for tracking. OpenXR (Khronos Group, 2025) is relevant here only as broader ecosystem context: SteamVR is currently an OpenXR-conformant runtime, but VRrec itself should not be understood as a native OpenXR application. In practice, VRrec can record only those headsets, controllers, and trackers that are successfully exposed to it through the current SteamVR-based path, that is, through the software route by which device data become available to VRrec via SteamVR. The reference configuration validated in this paper comprised an HTC Vive Pro HMD, HTC Vive controllers, and HTC Vive trackers in a SteamVR environment (see “Stress tests”). Beyond that reference setup, official Valve documentation currently names the Valve Index, HTC Vive, Oculus Rift, and Windows Mixed Reality headsets as supported SteamVR-side PC-VR families (Valve Corporation, 2023). Valve’s official Steam Link documentation additionally names Meta Quest 2, Meta Quest 3, and Meta Quest Pro for VR streaming from a computer running Steam and SteamVR (Valve Corporation, 2026b). For those Meta Quest devices, the official Steam Link route requires the Steam Link app on the headset plus a Windows PC running Steam and SteamVR. If users instead choose Meta’s own Meta Horizon Link PC-VR path, the Meta Horizon Link app must also be installed on the Windows computer (Meta Store Support, 2025). These broader headset families are listed here as officially documented platform-side compatibility examples rather than as individually validated VRrec test configurations.

**Fig. 1:**
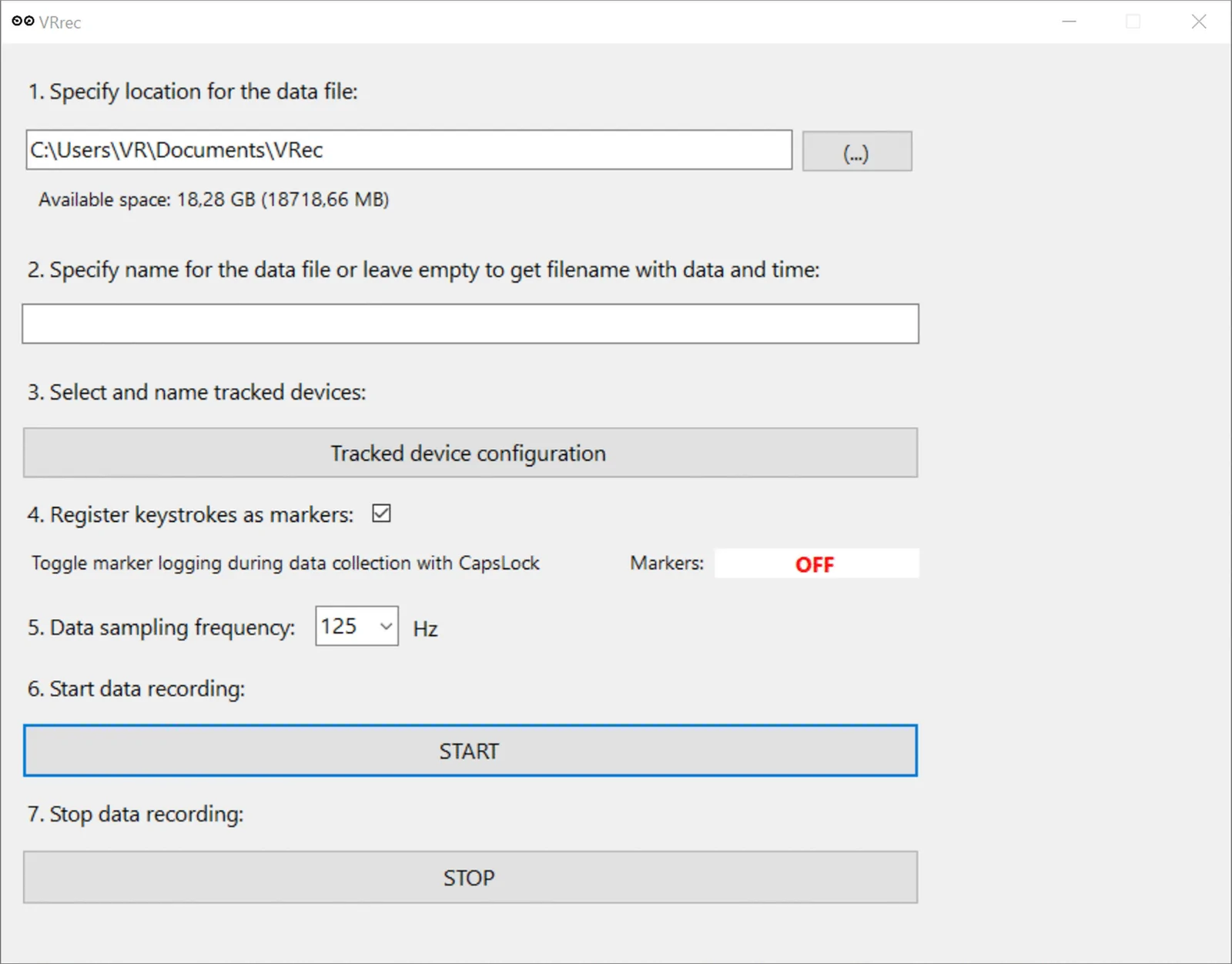
The main VRrec window with core application settings and *START/STOP* controls

**Fig. 2:**
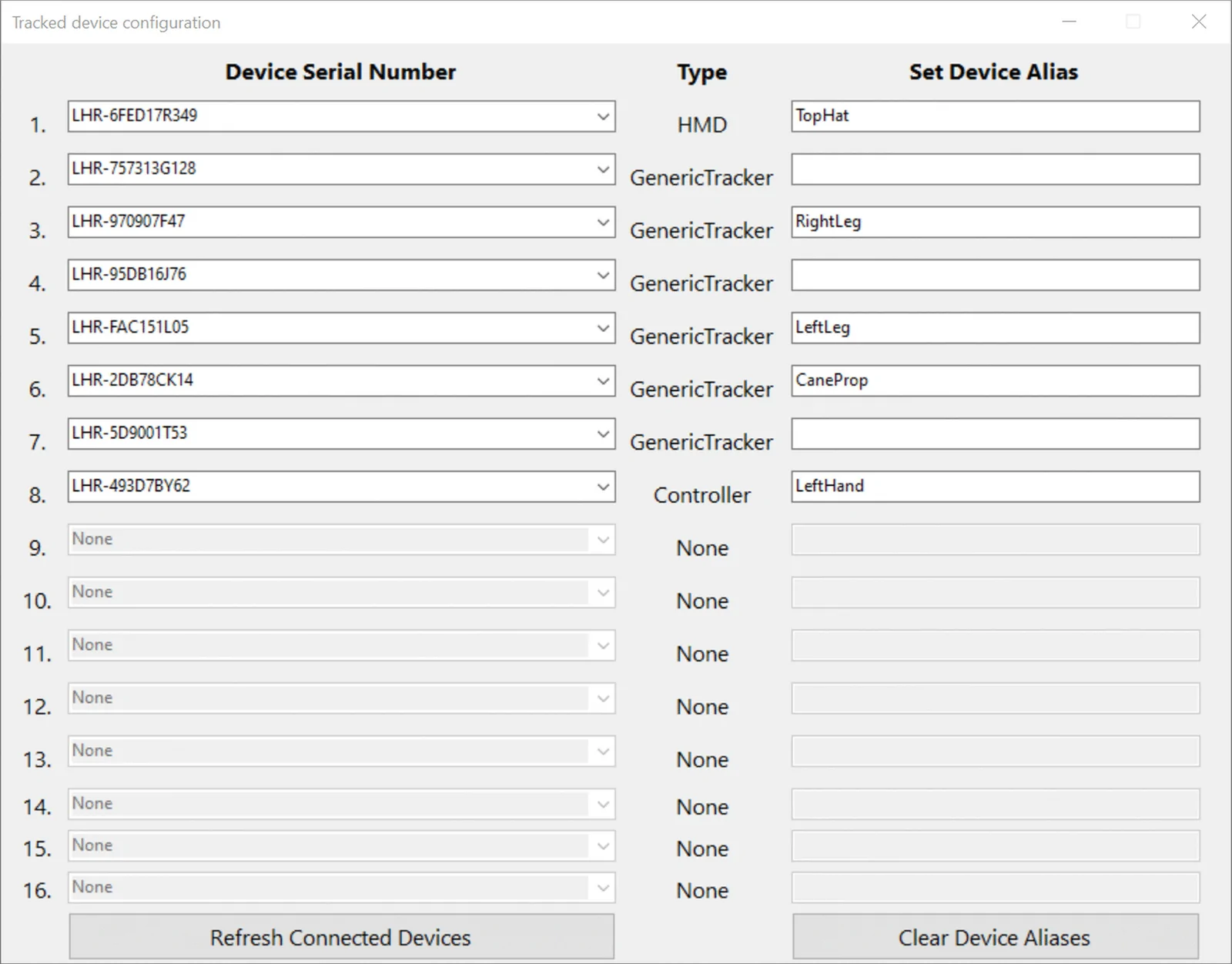
The device configuration window for naming and managing each tracked device recognized by VRrec

### Functionalities

VRrec performs a straightforward function: it records all movement data to a text file. To streamline this process, a set of additional functionalities has been implemented:

#### Choosing the location for the data file

The user must specify the drive and directory where the file should be saved. After selecting a directory, the system displays information on available disk space. If no directory is specified, the file is saved to the default location, i.e., Documents

VRrec.

#### Specifying the data file name

The user must provide a name for the data file to be saved. If no name is provided, the file is named with a timestamp in the “yyyy-MM-dd HH-mm-ss” format.

#### Selecting and naming tracked devices

By clicking the *Tracked device configuration* button, users can view the serial numbers of all tracked devices currently made available to VRrec through its current OpenVR/SteamVR path, including the head-mounted device (HMD), controllers, and trackers. Users can also assign an alias to each device, making specific trackers easier to identify during data analysis.

#### Sending markers

Any key on the keyboard can be used to insert a marker into the data. The key name is stored with a timestamp alongside all movement data. One restriction applies: to prevent accidental key logging, markers are recorded only when *Caps Lock* is on, a status indicated in the VRrec interface.

#### Selecting the data sampling frequency

Users can select the nominal data sampling frequency directly in the main VRrec window. The available options are 30, 60, 90, 125, 250, and 500 Hz.

#### Starting the data recording process

Pressing the *START* button begins the data-recording process. All positional and rotational data are saved as a text file in the specified directory.

#### Stopping the data recording process

Pressing the *STOP* button terminates the data-recording process and finalizes the data file. Device configuration and names remain unchanged, provided no device is disconnected.

### Graphical user interface

The VRrec graphical user interface (GUI) is designed to be both intuitive and minimalistic. On the main application window (Fig. 1), users can specify file-saving preferences, monitor available disk space, and quickly initiate or terminate data recording via the *START* and *STOP* buttons. Pressing *Caps Lock* switches key logging to ON (allowing markers to be recorded) or OFF (preventing markers from being recorded). The GUI clearly reflects this status, ensuring that VRrec only logs keystrokes when the user intends to do so. A prominent *Tracked device configuration* button opens a secondary configuration window (Fig. 2), allowing researchers to name and enable specific VR devices. Together, these two windows provide seamless control over essential options – such as data file destinations, naming conventions, and device selection.

## VRrec data output

### Technical data-output description

The VRrec application logs motion-capture data, event markers, and basic system information into a tab-separated values (TSV) text file. Each row in this file corresponds to one recorded sample (i.e., one “frame” of data), which includes multiple groups of columns for various connected devices (headset, controllers, or auxiliary trackers). These columns share a consistent naming scheme that indicates both the data category (e.g., System, Marker, HMD) and the type of value recorded (e.g., Bool, Float, String). Below, we summarize the principal data categories and illustrate how individual data fields are structured and interpreted.

#### File structure and naming convention

##### Rows

Each row corresponds to a single measurement. Measurements are taken at a sampling rate the user sets directly in the GUI (Fig. 1). Every row begins with system-level event information (the EventNumber and timestamp), followed by fields that capture the status and the positional and rotational parameters of each recognized VR device.

##### Column names

Each column name follows a descriptive pattern that indicates the data category, the device alias (optional), serial number, parameter name, and data type. For instance, the column named

Controller_LeftHand_LHR-493D7BY62_PosX_Float

belongs to the Controller category, references a device whose alias is LeftHand, serial number is LHR-493D7BY62, captures the *X*-coordinate of that device’s position, and stores it as a floating-point number, while

GenericTracker_LHR-75733G128_PoseValid_Bool

belongs to the GenericTracker category, references a device without an alias but whose serial number is LHR-75733G128, captures that device’s pose-validation status, and stores it as a Boolean value.

##### Device-specific fields

VRrec dynamically creates columns for each recognized device at the time of recording. Consequently, if multiple devices of the same type are connected (e.g., two trackers or several controllers), each device’s data are logged under its unique serial number and/or alias. The naming scheme remains consistent across devices, ensuring that, for example, PosX always refers to that device’s position on the *X*-axis.

##### Data categories, parameter names, and data types

System

- EventNumber (Uint): A sequential integer that increments each time a new row of data is recorded. This is useful for quickly indexing or filtering rows. The column name is: System_EventNumber_Uint
- DateTime (DateTime): A timestamp encoding the date and time of measurement. The format typically follows DD/MM/YYYY HH:mm:ss.fff, providing millisecond precision. Researchers can use this to align VRrec data with external data sources (e.g., biometric or behavioral logging) or to calculate interevent intervals. The column name is: System_DateTime_DD/MM/YYYY HH:mm:ss.fff It is important to note that the date separator (“/”) and the time separators (“:” and “.”) in the data values – but not in the column name itself – are localized according to the regional settings of the operating system running VRrec.

Marker

- Marker_Number (Uint): If a marker (i.e., an experimental event) is inserted manually during recording, this column stores the numeric label of that marker. The number is incremented by one each time a marker is inserted. The column name is: Marker_Number_Uint
- Marker_Data (String): A text field that captures the marker’s symbolic label. For instance, pressing a C-letter key records C in this field, whereas NoMarker appears when no key is pressed for that sample. The column name is: Marker_Data_String

HMD Data fields with the HMD prefix represent information recorded from the VR headset. The serial number and/or alias are appended (e.g., HMD_LHR-6FED17R349).

- Connection and validity
  - Connected (Bool): Indicates whether the headset is currently connected (True/False). An example column name: HMD_LHR-6FED17R349_Connected_Bool
  - PoseValid (Bool): Indicates whether device tracking is valid and usable. An example column name: HMD_LHR-6FED17R349_PoseValid_Bool
- Velocity
  - VelocityX, VelocityY, VelocityZ (Float): Linear velocity along each axis of headset movement. An example column name: HMD_LHR-6FED17R349_VelocityX_Float
  - AngularVelocityX, AngularVelocityY, AngularVelocityZ (Float): Rotational velocity around each axis (expressed in radians per second). An example column name: HMD_LHR-6FED17R349_AngularVelocityZ_Float
- Position
  - PosX, PosY, PosZ (Float): Three-dimensional coordinates of the headset in the VR tracking space, with the Y-axis indicating the vertical dimension. An example column name: HMD_LHR-6FED17R349_PosY_Float
- Orientation
  - RotX, RotY, RotZ, RotW (Float): Components of the device’s orientation in quaternion form (see “Position and orientation in space in a VR environment”). An example column name: HMD_LHR-6FED17R349_RotW_Float
- Transformation matrix
  - Matrix11 through Matrix34 (Float): A 3×4 transformation matrix that fully describes the device’s position and orientation in one compact representation. Each Matrix < R >< C > field corresponds to the matrix position denoted by row < R > and column < C > . While quaternions and positions are typically used for simpler motion analyses, these matrix components can be leveraged when advanced transformations or inverse kinematics are required. An example column name: HMD_LHR-6FED17R349_Matrix21_Float

Controller Column names labeled Controller mirror those in the HMD category. They include the same core set of fields (e.g., Connected_Bool, PoseValid_Bool, PosX_Float, RotX_Float, etc.) to reflect each controller’s state, velocity, position, and orientation. Example column names:

Controller_LeftHand_LHR-493D7BY62_PoseValid_Bool

Controller_LeftHand_LHR-493D7BY62_AngularVelocityX_Float

Controller_LeftHand_LHR-493D7BY62_Matrix32_Float

< Auxiliary Tracking Devices > Any additional tracker recognized by the system (e.g., a Vive Tracker or other specialized device) is logged with a prefix derived from its alias and/or default label (e.g., Tracker_LHR-75733G128). The same position, orientation, and velocity fields appear under this heading, providing a standardized record for each trackable object. Example column names:

Tracker_LHR-75733G128_PosZ_Float

Tracker_LHR-75733G128_RotX_Float

Tracker_LHR-75733G128_Matrix14_Float

#### Interpreting and using the data

Because each row in the TSV file contains every active device’s state at a unique timestamp, researchers can reconstruct a detailed timeline of the entire recording session. Combining system, marker, and device-specific information in one unified file enables straightforward synchronization and analysis in post-processing software (e.g., Python, R, or MATLAB). Users may filter or segment the data by Marker_Number and Marker_Data to isolate particular experimental events, examine head and controller motions around those events, or correlate them with external measures such as physiological signals.

In sum, this data structure is designed to maximize flexibility and clarity for subsequent analysis. Each device is clearly identified by its serial number and/or user-defined alias, and each motion or status parameter is labeled in a consistent, self-explanatory manner.

### Position and orientation in space in a VR environment

#### Position

The term “position” refers to the absolute location in 3D space where a given device (headset, controller, tracker) is situated. It is described by three values representing coordinates in a specific reference coordinate system (LaValle, 2023). Although there are multiple 3D coordinate systems, VR technology generally adopts the Cartesian system with the *X*, *Y*, and *Z* axes.

**Fig. 3:**
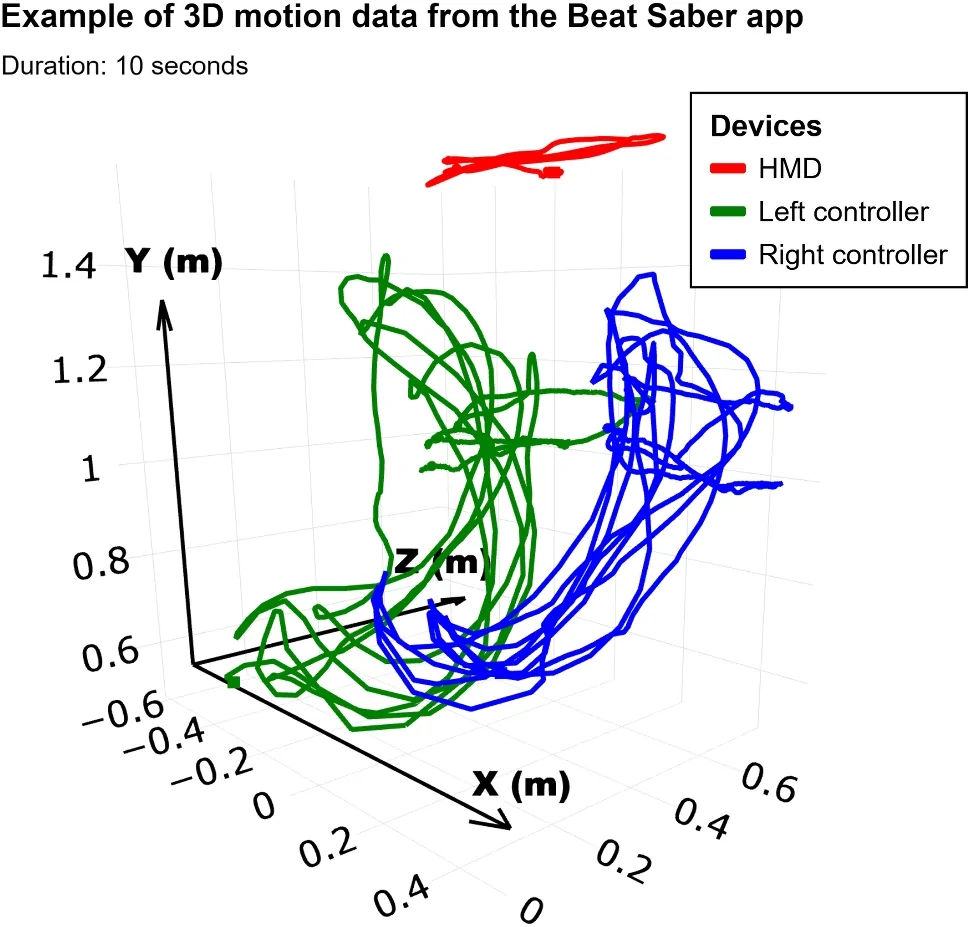
Example of 3D motion data from the Beat Saber app

**Fig. 4:**
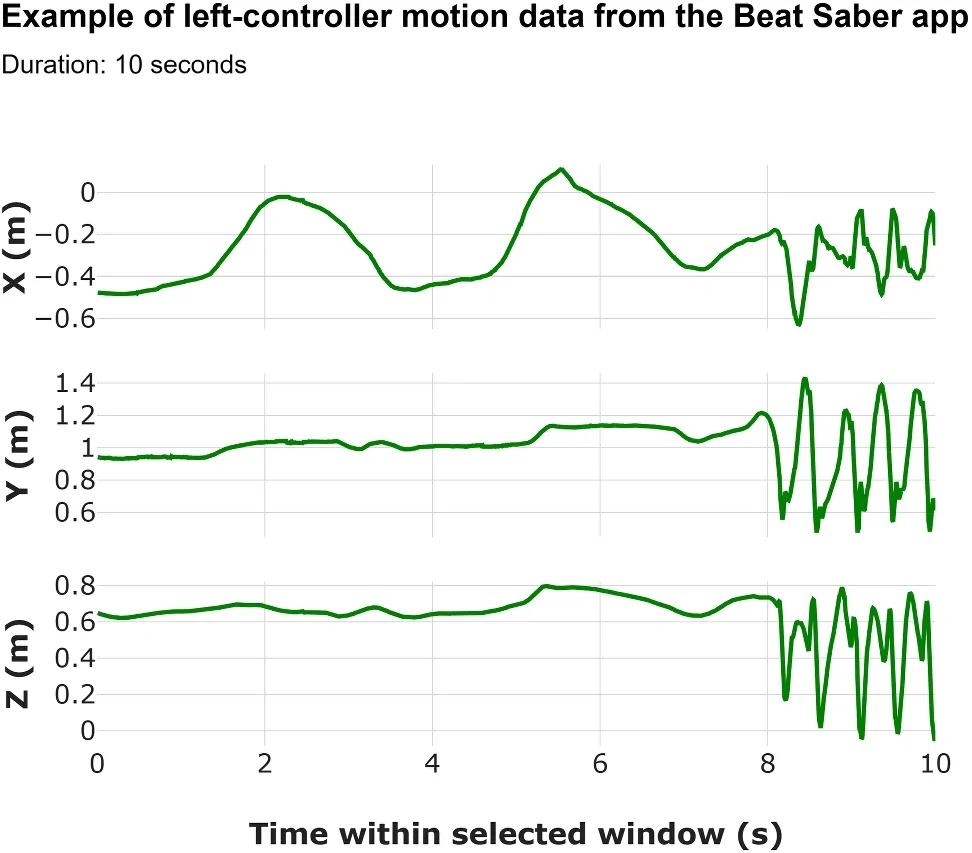
Example of left-controller motion data from the Beat Saber app

To help readers understand what VRrec positional data can look like in practice, Figs. 3 and 4 present a short example from a Beat Saber session – a fast-paced rhythm-based VR game (Beat Games S.R.O., 2018). Figure 3 shows the 3D trajectories of the HMD and both controllers, making visible the sweeping, cutting hand movements and slight side-to-side head movement, whereas Fig. 4 shows the left controller’s *X*, *Y*, and *Z* position signals from the same 10-s window. Both figures were generated from the example VRrec data-output file available in the open project repository.

#### Orientation

The term “orientation” denotes rotation of a given device relative to a reference orientation (LaValle, 2023). There are several ways to define orientation. The most intuitive is the Euler representation, which describes rotation using three angles about the *XYZ* axes. The order in which the rotations are applied is crucial, leading to multiple variations of this representation. Although Euler angles are easy to understand, they become ambiguous in two specific orientations, where one degree of freedom is lost, making it impossible to define the rotation angles uniquely. This phenomenon is known as *gimbal lock* (LaValle, 2023).

An alternative, though less intuitive, representation avoids gimbal lock by using quaternions (Kuipers, 2020; LaValle, 2023) – ordered sets of four numbers labeled *x*, *y*, *z*, and *w*. Geometrically, *x*, *y*, and *z* define a unit vector $\vec {a}=(u_x, u_y, u_z)$ about which the object is rotated, whereas *w* is related to the rotation angle θ. The values *x*, *y*, *z*, and *w* are normalized in a specific way.

The component *w* is defined as the cosine of half the rotation angle, whereas *x*, *y*, and *z* correspond to the components of the unit vector $(u_x, u_y, u_z)$ multiplied by the sine of half that angle:

1 $$\begin{aligned} w= &  \cos {\frac{\theta }{2}}, \end{aligned}$$

2 $$\begin{aligned} x= &  u_x\sin {\frac{\theta }{2}}, \end{aligned}$$

3 $$\begin{aligned} y= &  u_y\sin {\frac{\theta }{2}}, \end{aligned}$$

4 $$\begin{aligned} z= &  u_z\sin {\frac{\theta }{2}}. \end{aligned}$$

Mathematically, a quaternion can be considered an extension of complex numbers with additional imaginary components:

5 $$\begin{aligned} q=w+ix+jy+kz, \end{aligned}$$

where *i*, *j*, and *k* are imaginary units that form an orthogonal basis in the Cartesian reference frame. Consequently, quaternions support algebraic operations such as addition, multiplication, and division.

In VR technology, which is closely related to computer graphics, rotating an object involves transforming all its vertices (points that define its geometric mesh) by a given angle θ about a vector $\vec {a}$. In practice, quaternion-based rotation is performed as follows:

1. Represent the point $p=(p_x,p_y,p_z)$ as a purely imaginary quaternion: $p=ip_x+jp_y+kp_z$
2. Express the rotation using a quaternion as defined above.
3. Compute the new position of *p* after rotation by angle θ about vector $(u_x,u_y,u_z)$ using the conjugation operation: $p'=qpq^{-1}$

Quaternion algebra requires the following multiplication rules:

6 $$\begin{aligned} i^2 = j^2 = k^2 = ijk = -1 \end{aligned}$$

and an extended form of Euler’s formula:

7 $$\begin{aligned} q=e^{\frac{\theta }{2}(iu_x+ju_y+ku_z)}=\cos {\frac{\theta }{2}}+(iu_x+ju_y+ku_z)\sin {\frac{\theta }{2}} \end{aligned}$$

These operations enable straightforward execution of complex rotation sequences:

8 $$\begin{aligned} q=\dots q_4q_3q_2q_1 \end{aligned}$$

Unfortunately, because of the complex and unintuitive nature of quaternions, we do not include a separate illustration of raw quaternion values. Unlike the positional data shown in Figs. 3 and 4, quaternion values do not provide directly readable angle coordinates. Because quaternions form a normalized 4D representation with one real component and three imaginary components, plotting their raw values does not yield a trajectory that can be interpreted intuitively in visual terms. In practice, readers can infer more about body orientation from the mutual spatial arrangement of the HMD and controllers in Fig. 3 than from raw quaternion-component traces.

## Stress tests

To assess VRrec’s resilience and reliability under extended operation and demanding usage scenarios, we conducted two types of stress tests. The first aimed to examine performance when a fully interactive VR application was running, while the second investigated system stability and data fidelity in a continuous, long-term recording session.

### Demanding scenario

We carried out ten individual sessions in which a participant continuously used a VR system with VRrec running in the background. Each session lasted 2 h, for a total of 20 h of active VR usage. Beat Saber (Beat Games S.R.O., 2018) was run concurrently, placing additional computational and tracking demands on the hardware and software. The tracked devices included one HTC Vive Pro HMD, two HTC Vive controllers, and six HTC Vive trackers placed on the body of the VR user, all operating in a SteamVR environment (Valve Corporation, 2023).

Throughout these 2-h sessions, VRrec maintained continuous motion capture without crashes or observable data interruptions. The tracking data (positional and rotational) were written to the output file in real time, and keyboard-inserted event markers were logged accurately. This demonstration of stability suggests that VRrec can reliably support even motion-heavy VR experiences without data loss or application failure.

### Long-term scenario

To examine VRrec’s behavior in a prolonged scenario, we conducted a 24-h stress test, which – due to its extreme duration – had to be performed without a user. In this setup, to ensure uninterrupted operation, the VR devices (HMD, controllers, and trackers) were connected to their power adapters and kept stationary. SteamVR’s default home environment was left running continuously, while VRrec recorded motion capture data – albeit with no real participant movement – throughout the day.

No software failures were detected during the 24-h interval. VRrec continued to log data at the specified sampling rate until manually stopped. As in the active-user test, the data files contained consistent streams of motion data, confirming the application’s capacity for extended-duration recording.

### Data validation

After both stress tests, the recorded outputs were checked with a dedicated validation script, written in the R programming language and included in the project repository for readers and future users. A full description of the script’s functionality and operating instructions is beyond the scope of the article, but both are provided directly in the script file as technical comments. This script was a central part of the validation workflow: it allowed us to verify that VRrec output files could be parsed structurally and to summarize the exported variables technically and statistically. Specifically, the script imports a selected VRrec export, saves any parsing-problem rows if they are detected, derives inter-sample timestamp differences and corresponding effective sampling frequencies, converts pose-derived motion values to missing values whenever the matching device-level PoseValid flag is false (see “File structure and naming convention”), and saves per-variable inspection tables with descriptive statistics and missing-data information for the exported VRrec parameters. The validation runs reported here produced no parsing-problem data rows in either the 24-h stationary-device stress test or the Beat Saber demanding stress-test files.

Because the 24-h stress test produced a large output file of approximately 30 GB, we divided it into 24 approximately hourly parts before applying the R validation script. The validation evidence therefore consisted of 24 files from the long-term scenario and ten files from the demanding scenario.

The timestamp summaries showed that the central effective sampling pattern was stable. For the 24 approximately hourly long-term files, quantile summaries were computed separately for each part and were identical across all 24 parts: the median inter-sample interval was 8 ms, the exact 8-ms value extended from the 5th to the 95th percentile, and the 0.5th-to-99.5th percentile range was 7–9 ms. For the ten Beat Saber stress-test files, the median was also 8 ms in every file and the 0.5th-to-99.5th percentile range was 7–9 ms in every file; the exact 8-ms value extended at least from the 15th to the 85th percentile in all ten files, and from the 5th to the 95th percentile in 7 of 10 files. These results are consistent with the nominal 125-Hz sampling setting. The same summaries also exposed rare edge outliers. In the 24-h stationary-device test, every approximately hourly part contained at least one 0-ms timestamp difference, and the largest part-level maximum was 752 ms; in the Beat Saber stress-test files, the largest maximum was 34 ms. The 0-ms values should be interpreted in light of the timestamp representation: timestamps expressed in seconds are stored to three decimal places, giving millisecond-level resolution with an uncertainty of approximately ±1 ms, so a reported 0-ms difference can represent a nonzero interval shorter than 1 ms after rounding rather than a truly zero-duration interval. Together with the large maximum values, these very short intervals indicate occasional delays and subsequent catch-up bursts, in which delayed samples were followed by very short inter-sample intervals. In practical terms, timer events that could not be handled during a delay may then be delivered to VRrec in a batch. The available output does not allow us to assign these edge events uniquely to VRrec, the operating system, disk I/O, or the upstream VR stack. Importantly, however, VRrec did not crash or hide these irregularities. It recorded them transparently, and the saved output plus the validation R script made the timing pattern diagnosable after recording.

The same validation workflow was also used to screen the stationary 24-h recording for long-term positional drift. Because the script produced inspection summaries for every exported variable in each approximately hourly part, we could compare the hourly medians of every positional variable for every tracked device. These PoseValid-aware hourly medians did not show any linear or nonlinear time trend across tracked devices; the long-term median trend was effectively absent. Even if such a trend had appeared, it would have had to be interpreted as a property of the upstream tracking technology, because VRrec records incoming data transparently and does not modify the motion values it receives. As for short-term substantial offsets, they were associated with validity-state changes in the upstream tracking data; this upstream-technology interpretation is discussed in the limitations section rather than treated as a property of the VRrec application or of the stress-test procedure itself.

## Limitations

Although VRrec addresses an important need for a zero-coding, open-source body-motion capture tool within PC-based VR research, several limitations must be acknowledged. Some of these limitations stem from VRrec itself as a background recorder, whereas others stem from the upstream technologies and deployment conditions from which VRrec receives data.

First, VRrec currently allows event markers to be inserted only manually via the computer keyboard, which limits precise or automated marker insertion. In future versions, the authors or the open-source community could consider additional marker-input channels, such as a simple network socket or USB serial input, to reduce this limitation.

Second, VRrec is intentionally designed to operate without direct access to the target application’s source code or internal state. As a result, event marking cannot be inserted from within the VR software itself. Researchers can insert event markers using only the keyboard. Consequently, investigators must rely on manual or external means of synchronization between VR content events (such as scene changes, stimuli onsets, or interactions) and the recorded body-motion data. For the same reason, research questions that depend on direct knowledge of distances to dynamic virtual objects or avatars are not directly supported by VRrec alone. In some constrained cases, however, researchers may partially approximate such measures indirectly, for example by calibrating the locations of static objects before data collection, placing an additional tracker at a known fixed spatial reference, or analyzing participant position relative to a known axis or condition structure. Such strategies remain researcher-dependent and do not provide native access to dynamically changing application states.

Third, VRrec focuses exclusively on data capture. It does not provide built-in analytics or data-processing capabilities. Researchers wishing to compute specific behavioral metrics, detect patterns in the motion data, or conduct complex statistical analyses must do so using additional software and often custom scripting in languages such as Python or R. While VRrec aims to eliminate coding requirements for data acquisition, it does not obviate the need for programming expertise (or analysis-focused tools) when researchers perform advanced data interpretation.

Fourth, VRrec requires a Windows PC environment and the SteamVR platform to track devices. Accordingly, users should understand the present implementation as requiring SteamVR-based PC VR through the OpenVR path used by VRrec, rather than direct use of arbitrary OpenXR runtimes. Additional PC-VR headsets may work when SteamVR exposes them successfully, but such cases should be understood as SteamVR-mediated compatibility (“System requirements and compatibility”) beyond the paper’s tested reference setup and should be pilot-tested before research use. This restriction inherently excludes applications running natively in a headset’s standalone environment. Standalone-capable headsets such as Meta Quest can participate only when switched into a PC-VR path that uses a Windows computer. In that case, VRrec records motion from the PC-side VR application rather than from apps running directly on the headset. As a result, VRrec’s utility is constrained to setups where a PC-based VR system is available. Researchers who rely exclusively on standalone VR cannot directly employ VRrec in their experiments.

A separate set of constraints concerns the upstream technologies and deployment conditions from which VRrec receives data. The practical quality of recorded measurements depends on the underlying tracking stack, hardware drivers, platform updates, calibration, and device configuration. Users should therefore pilot test VRrec in their target setup, verify that tracked devices are recognized correctly, and confirm that the recorded data quality is adequate for the intended analysis.

For example, prior work on the HTC Vive (HTC Corporation, 2018) has shown that consumer VR tracking can exhibit position and orientation offsets after tracking loss and recovery (Niehorster, Li, & Lappe, 2017). Additional analyses of the 24 hourly outputs from the stationary-device stress test are consistent with the experimental results obtained by those authors, although our stress test was not designed as a direct replication of Niehorster et al.’s controlled tracking-accuracy experiments. First, the percentage of invalid pose samples was strongly correlated with positional spread, for example Pearson r=.814 and Spearman ρ=.858 for the robust spread diagnostic based on the 99.5th minus 0.5th percentiles. Second, using a 1-cm position-deviation threshold, a complementary analysis showed that anomaly windows within 5 s of PoseValid = FALSE runs (see “Data categories, parameter names, and data types”) were much more frequent than in false-free baseline windows (90.579% vs. 1.562%), and false-run events were much more likely than false-free control anchors to have a 1-s pre/post anomaly (99.900% vs. 2.265%). Together, these results suggest that the stationary-test offsets are best interpreted as consequences of the HTC Vive tracking setup and its loss/recovery behavior rather than as failures of VRrec as a recorder.

This interpretation follows directly from VRrec’s intended function. VRrec does not transform, smooth, or correct the incoming upstream tracking stream; it records the stream and associated status fields exposed by the VR stack. In the stress tests, this transparency was useful: even when upstream tracking-state problems appeared, VRrec remained operational, continued writing the incoming data, and preserved timing, device-state, missingness, and the column structure of the motion-data table in the output file, including cases in which a tracker or a controller disconnected for any reason, such as battery depletion, in a form that allowed the issues to be diagnosed and reconstructed.

As for the timing results discussed earlier in “Data validation”, which may also reflect limitations of upstream technologies, they should be interpreted in light of the fact that many human body-motion analyses do not require millisecond-level timing precision. Whole-body, head, and controller movements unfold at lower frequencies than signals such as eye movements, and the analytical needs that follow from these signals are typically different unless the study is designed specifically to measure reaction times or similarly time-critical events. VRrec should therefore be understood as a transparent motion-data recorder with inspectable timing information, without the ability to perform millisecond-accurate reaction-time measurement inside arbitrary third-party VR applications.

## Conclusion

VRrec addresses a critical need in VR-based behavioral research by simplifying body-motion capture and eliminating the requirement for researchers to write code or build the target VR application in order to record motion data. By operating discreetly in the background, VRrec allows researchers to use off-the-shelf third-party VR software across a wide variety of experimental protocols, thus reducing technical barriers while preserving experimental flexibility. The tool’s open-source framework further encourages community-driven enhancements and fosters transparency, making it adaptable to diverse fields ranging from social psychology to rehabilitation. We hope that VRrec itself will enable more numerous and varied studies and that this article will also inspire further innovations and breakthroughs in VR-based behavioral science.

## Data Availability

Not applicable.

## Code availability

The source code and executable version of the VRrec tool, the validation script written in the R programming language for inspecting VRrec output files, and an example data-output file are available at https://github.com/OPI-PIB/VRrec. The example data-output file includes the data used to generate Figs. 3 and 4.
